# Use of Chimeras, Point Mutants, and Molecular Modeling to Map the Antagonist-binding Site of 4,4′,4″,4‴-(Carbonylbis-(imino-5,1,3-benzenetriylbis(carbonylimino)))tetrakisbenzene-1,3-disulfonic Acid (NF449) at P2X1 Receptors for ATP*

**DOI:** 10.1074/jbc.M114.592246

**Published:** 2014-11-25

**Authors:** Louise K. Farmer, Ralf Schmid, Richard J. Evans

**Affiliations:** From the Departments of ‡ Cell Physiology and Pharmacology and; § Biochemistry, University of Leicester, Leicester LE1 9HN, United Kingdom

**Keywords:** ATP, Electrophysiology, Ion Channel, Mutagenesis, Purinergic Receptor, NF449, P2X Receptor, PPADS, Ligand-gated Ion Channel, Suramin

## Abstract

P2X receptor subtype-selective antagonists are promising candidates for treatment of a range of pathophysiological conditions. However, in contrast to high resolution structural understanding of agonist action in the receptors, comparatively little is known about the molecular basis of antagonist binding. We have generated chimeras and point mutations in the extracellular ligand-binding loop of the human P2X1 receptor, which is inhibited by NF449, suramin, and pyridoxal-phosphate-6-azophenyl-2,4-disulfonate, with residues from the rat P2X4 receptor, which is insensitive to these antagonists. There was little or no effect on sensitivity to suramin and pyridoxal-phosphate-6-azophenyl-2,4-disulfonate in chimeric P2X1/4 receptors, indicating that a significant number of residues required for binding of these antagonists are present in the P2X4 receptor. Sensitivity to the P2X1 receptor-selective antagonist NF449 was reduced by ∼60- and ∼135-fold in chimeras replacing the cysteine-rich head, and the dorsal fin region below it in the adjacent subunit, respectively. Point mutants identified the importance of four positively charged residues at the base of the cysteine-rich head and two variant residues in the dorsal fin for high affinity NF449 binding. These six residues were used as the starting area for molecular docking. The four best potential NF449-binding poses were then discriminated by correspondence with the mutagenesis data and an additional mutant to validate the binding of one lobe of NF449 within the core conserved ATP-binding pocket and the other lobes coordinated by positive charge on the cysteine-rich head region and residues in the adjacent dorsal fin.

## Teaser

**Background:** NF449 is a selective P2X1 receptor antagonist.

**Results:** Chimeric/mutant P2X1/4 receptors and molecular docking provided models of NF449 binding.

**Conclusion:** NF449 is coordinated by the core ATP-binding site, the base of the cysteine-rich head, and the dorsal fin region.

**Significance:** The study provides insight into the selective antagonism of P2X receptors that may aid rational drug design.

## Introduction

P2X receptors comprise a family of ATP-gated ion channels made from the homo- and heterotrimeric assembly of seven receptor subunits (P2X1–7) with intracellular N and C termini, two transmembrane-spanning segments, and a large extracellular ligand-binding loop (1). In the 20 years since the receptors were first cloned (2, 3), the roles of defined P2X receptor subtypes in a range of physiological processes and pathophysiological conditions have been determined (4, 5). This has highlighted the therapeutic potential of P2X receptor subtype-selective antagonists. One target is the P2X1 receptor that is expressed on platelets; receptor knock-out mice had normal bleeding responses but were protected from thrombosis (6), and overexpression of the receptor in platelets was prothrombotic (7). The P2X1 receptor-selective antagonist 4,4′,4″,4‴-(carbonylbis(imino-5,1,3-benzenetriylbis(carbonylimino)))tetrakisbenzene-1,3-disulfonic acid (NF449)^2^ had no effect on normal bleeding in mice, but reduced platelet aggregation in a model of thromboembolism and reduced thrombus size following laser-induced injury of mesenteric arterioles (8). Thus, P2X1 receptor-selective antagonists would protect from thrombosis, heart attack, and stroke without affecting normal hemostasis.

The crystallization of the zebrafish P2X4 receptor in an ATP-bound conformation provided structural insight into the agonist-binding site at P2X receptors (9) and is supported by a range of mutagenesis studies (10–13). The P2X receptor subunit structure resembles a dolphin with a cysteine-rich head, body, flippers, dorsal fin, and transmembrane fluke (14). ATP binds at the intersubunit interfaces in pockets formed by the head, upper body, and left flipper of one subunit with the lower body and dorsal fin of the adjacent one (9). However, there is currently little or no structural information on the site(s) of antagonist binding at P2X receptors. Previous studies highlighted roles of residues around the agonist-binding pocket involved in the action of the relatively nonselective P2X receptor antagonists suramin and pyridoxal-phosphate-6-azophenyl-2,4-disulfonate (PPADS) (15–17). For the P2X1 receptor-selective antagonist NF449, mutagenesis-based approaches identified positive charges at the base of the cysteine-rich head region, adjacent to the agonist-binding site, as contributing to high affinity binding (18, 19). However, the full extent of the binding site remains to be determined. In this study, we used chimeras to assess the contribution of variations in the extracellular loop to antagonist action. To optimize the approach, we replaced sections of the extracellular loop of the human P2X1 (hP2X1) receptor with the corresponding parts of the rat P2X4 (rP2X4) receptor, which is essentially insensitive to the antagonists NF449, suramin, and PPADS (15, 20–22). Site-directed mutagenesis was then used to identify individual residues that contribute to NF449 action, which were the starting point for *in silico* antagonist docking and validation.

## EXPERIMENTAL PROCEDURES

### 

#### 

##### P2X Receptor Constructs and Generation of Chimeras and Point Mutants

The hP2X1 receptor cDNA was originally cloned from the bladder (23), and the rP2X4 receptor DNA was a gift from Dr. Francois Rassendren (CNRS, Montpellier, France). A mutation (Y378A) was introduced to the rP2X4 receptor template to give more stable and reproducible currents (24). To design chimeras, regions of conservation of three or more residues between the sequences of the hP2X1 and rP2X4 receptors were identified using a sequence lineup that was generated using the protein BLAST program (NCBI) and manually corrected. Five of these conserved regions were chosen as borders to four initial sections (A–D) that were swapped between the receptors. Four of these were 100% conserved and easy to choose as borders to regions A–D using the lineup. There was no obvious island of conservation in a suitable location to form the border between regions C and D, so a section of six amino acids with >65% conservation located around the conserved Cys-261 was chosen. The initial four regions swapped consisted of residues 56–132 in region A, residues 133–184 in B, residues 185–261 in C, and residues 262–330 in D. Chimeras were generated by domain-swapping PCR as described previously (25). Point mutations were made using the QuikChange mutagenesis kit (Stratagene). Mutations were verified by DNA sequencing (Protein and Nucleic Acid Chemistry Laboratory Services, University of Leicester).

The original chimera replacing region A (residues 56–132) was nonfunctional due to a lack of expression at the cell surface. This was then redesigned to exclude residues closest (56–62) to transmembrane domain 1 from the swapped region. These residues were not located at the surface of the receptor and were therefore unlikely to be involved in NF449 binding. The region A chimera (X1-AX4) referred to here consists of the hP2X1 receptor with residues 63–133 of the rP2X4 receptor. The mean peak current evoked to a maximal concentration of ATP was the same for the WT and all mutant receptors, with the exception of the X1-AX4 chimera, which had an ∼5-fold reduction in peak current compared with the hP2X1 receptor (*p* < 0.0001).

##### Expression in Xenopus laevis Oocytes

cRNA was synthesized using the T7 mMESSAGE mMACHINE kit (Ambion) and injected into stage V *X. laevis* oocytes as described previously (23). Oocytes expressing P2X receptors were stored at 16 °C in ND96 buffer (96 mm NaCl, 2 mm KCl, 1.8 mm CaCl_2_, 1 mm MgCl_2_, 5 mm sodium pyruvate, and 5 mm HEPES, pH 7.6) with 50 μg/ml gentamycin for 3–7 days. For electrophysiological recordings, gentamycin was not present, and 1.8 mm CaCl_2_ was replaced with 1.8 mm BaCl_2_.

##### Electrophysiological Recordings

Two-electrode voltage clamp recordings were carried out using a GeneClamp 500B amplifier with a Digidata 1322A analog-to-digital converter and pCLAMP 8.2 acquisition software (Molecular Devices, Menlo Park, CA) at a holding potential of −60 mV. ATP (Mg^2+^ salt, Sigma) was applied via a U-tube perfusion system for 3 s at 5–10-min intervals (dependent on the P2X receptor) to allow for reproducible responses to be recorded. Antagonists were bath-perfused in ND96 buffer for 5 min before they were co-applied with an EC_90_ of ATP through the U-tube. To generate inhibition curves, antagonists were co-applied with an EC_90_ of ATP to standardize any shift in ATP potency. Antagonists fully equilibrated with the receptor during the first application period as the level of inhibition was maintained on a second test application. The inhibitory effects of the antagonists were reversed in the washout period between agonist applications. Antagonists (at maximal concentrations used) were applied to the WT and all mutant receptors in the absence of ATP and were seen to have no effect on the holding current. Suramin was from Sigma, PPADS from Tocris, and NF449 from Abcam.

##### Data Analysis

Individual normalized concentration-response curves were fitted with the Hill equation (variable slope) with GraphPad Prism 6 (GraphPad Software, San Diego, CA). For agonists, pEC_50_ is the −log_10_ of the concentration giving 50% maximal response (EC_50_ value), and IC_50_ is the concentration of antagonist inhibiting the EC_90_ of ATP by 50%. pIC_50_ is the −log_10_ of the IC_50_ value. For the calculation of EC_50_/IC_50_ values and Hill slopes, individual concentration-response curves were generated for each experiment, and statistical analysis was carried out on the data generated. In the figures, inhibition curves are fitted to the mean normalized data.

Any significant differences between the WT, chimeras, and mutants (*e.g.* current amplitude, rise and desensitization/current remaining at the end of the ATP application, Hill slope, pEC_50_, and pIC_50_) were calculated by one-way analysis of variance, followed by Dunnett's test. The software used was GraphPad Prism 6 (*n* ≥ 3 for all data points).

##### P2X1 Receptor Modeling and NF449 Ligand Docking

Homology models for the hP2X1 receptor were built based on the x-ray structure (Protein Data Bank ID 4DW0) of the zebrafish P2X4 receptor apo form as described previously (26). These models were used as receptors for ligand docking in GOLD (27). The antagonist NF449 was prepared for docking and energy-minimized in HyperChem v8.0 (Hypercube, Inc., Gainesville, FL). The docking site in the hP2X1 receptor models was set to sample the regions defined by point mutations in the cysteine-rich region and dorsal fin (Lys-136, Lys-138, Arg-139, Lys-140, Thr-216, and Gln-231). The receptor set-up treated lysine and arginine side chains within the docking site as flexible by using rotamer ensembles. Docking poses were generated using the genetic algorithm implemented in GOLD. The dimensionless Astex statistical potential score was used to rank the resulting NF449 docking poses. Poses with Astex statistical potential scores of >35 were visualized in PyMOL and further analyzed for their interactions with the six residues mentioned above. Four docking poses (poses A–D) showed interactions with most of the residues above and were used for further analysis.

##### Molecular Dynamics Simulations

The extracellular domains of the P2X1 models with NF449 in poses A–D were used as starting structures for molecular dynamics simulations. The molecular dynamics simulations were performed in AMBER 12 (28) with the ff99SB and GAFF force fields using AM1-BCC partial charges for NF449. Protein and antagonist were solvated in a cubic TIP3P water box and neutralized by adding counterions. Before the simulations, the systems were energy-minimized in three steps (constraints on non-hydrogen atoms, constraints on non-solvent atoms, and no constraints). The energy-minimized complexes were then equilibrated by heating for 50 ps from 0 to 300 K with weak restraints for all protein residues (2.0 kcal/mol Å^−2^), followed by density equilibration for 100 ps. Production runs were for 10 ns with 2-fs time steps. For all simulations, the SHAKE algorithm was used to constrain bonds between hydrogens and heavy atoms, and Langevin dynamics were used for temperature control. Trajectories were analyzed with the AMBER ptraj module.

## RESULTS

### 

#### 

##### Characterization of ATP Action at P2X1/4 Receptor Chimeras

**FIGURE 1.**
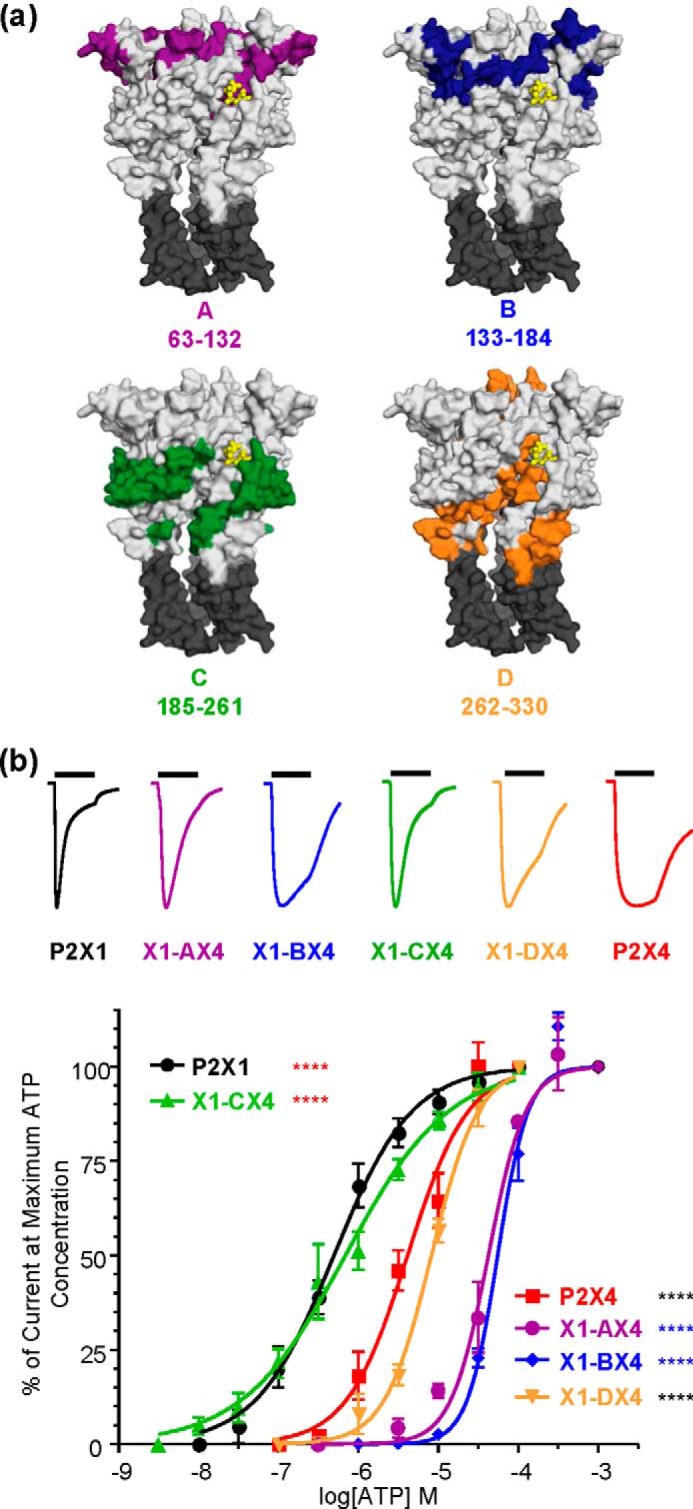
**Characterization of ATP at chimeric P2X receptors.** *a*, location of regions A–D on an ATP-bound homology model of the P2X1 receptor. Regions A–D are shown in color; ATP is shown in *yellow*. Transmembrane domains are shown in *dark gray. b*, *upper*, representative traces for WT and chimeric receptors at maximal ATP concentrations. The *bars* represent a 3-s ATP application. Traces have been normalized to peak currents to allow for comparison. *Lower*, concentration-response curves for ATP. *Black asterisks* indicate significant shifts in EC_50_ from the WT P2X1 receptor, *red asterisks* from the P2X4 receptor, and *blue asterisks* from both receptors. ****, *p* < 0.0001.

Four chimeras replacing sections of the extracellular loop of the hP2X1 receptor with the corresponding part of the rP2X4 receptor were designed with the aid of sequence lineups and homology models. Regions A–D each consisted of >50 residues; region C was the largest (76 amino acids), and region B was the smallest (51 amino acids). (The crossover points between regions were chosen to contain three to six conserved residues.) Region A contained residues 63–132, region B contained residues 133–184, region C contained residues 185–261, and region D contained residues 262–330 (hP2X1 receptor numbering). The nomenclature for the chimeras indicates the part replaced, *e.g.* X1-AX4 indicates the replacement of region A of the hP2X1 receptor with the corresponding region of the rP2X4 receptor. Of the residues in regions A, B, and D, ∼50% were conserved. Region C had more variance, with only 35% conservation. Each of the chimeras contained residues in or around the ATP-binding pocket. Regions A and B contained residues located mainly at the top of the receptor, above the ATP-binding pocket (Fig. 1). Region C was adjacent to and immediately below the ATP-binding pocket, and region D was mainly below the binding site, with some residues located at the apex of the receptor.

ATP evoked concentration-dependent currents at hP2X1 and rP2X4 receptors (EC_50_ ∼ 1 and 10 μm, respectively) that showed contrasting levels of desensitization to continued agonist application (19.7 ± 4.3% and 71.4 ± 4.5%, respectively, remaining at the end of a 3-s application of a maximal concentration of ATP), consistent with previous reports (15, 20, 21, 23). The hP2X1 receptor-based chimeras all gave robust reproducible responses to ATP (Fig. 1 and Table 1). For the X1-CX4 chimera, ATP potency (EC_50_ ∼ 1 μm) and desensitization (25.3 ± 2.9% current remaining at the end of a 3-s application of maximal ATP) were indistinguishable compared with the WT hP2X1 receptor. The X1-DX4 chimera showed rP2X4 receptor agonist sensitivity (EC_50_ ∼ 10 μm) with desensitization that was intermediate between the hP2X1 and rP2X4 receptors (40.1 ± 2.1% peak current remaining at the end of application, *p* < 0.01). ATP was ∼10-fold less potent at chimeras X1-AX4 and X1-BX4 (pEC_50_ = 4.43 ± 0.08 and 4.28 ± 0.04, respectively; *p* < 0.0001) than at the rP2X4 receptor (pEC_50_ = 5.39 ± 0.10). Interestingly, although these chimeras showed a similar large decrease in ATP potency compared with the WT hP2X1 receptor, the time course of X1-AX4 receptors (29.2 ± 11.4% current remaining) was the same as that for the WT hP2X1 receptor, whereas the time course of X1-BX4 was equivalent to that of the rP2X4 receptor (59.5 ± 2.9% and 71.4 ± 4.5% current remaining at the end of agonist application for X1-BX4 and rP2X4, respectively). These studies demonstrate a complex role of residues in the extracellular loop in determining responses to ATP and that agonist potency and time course can be regulated independently.

**TABLE 1:** **ATP and antagonist properties of region A–D chimeras** Antagonist properties were determined at the EC_90_ of ATP. ND, not determined; aa, amino acids.

	Peak current	ATP		Suramin		PPADS		NF449	
		pEC_50_	Current remaining at 3 s	pIC_50_	Hill slope	pIC_50_	Hill slope	pIC_50_	Hill slope
	*nA*		%						
P2X1	7598 ± 793	6.31 ± 0.12	19.7 ± 4.3	5.86 ± 0.06	1.14 ± 0.02	5.80 ± 0.02	1.26 ± 0.15	8.91 ± 0.07	1.23 ± 0.11
P2X4	5320 ± 586*^a^*	5.39 ± 0.10*^b^*	71.4 ± 4.5*^b^*	≪4.00	ND	≪4.00	ND	≪6.00	ND
X1-AX4 (aa 63–132)	1438 ± 340*^b^*	4.43 ± 0.08*^b^*	29.2 ± 11.4*^a^*	6.60 ± 0.14*^c^*	1.30 ± 0.22*^a^*	6.18 ± 0.02*^a^*	1.49 ± 0.08*^a^*	8.21 ± 0.02*^d^*	1.74 ± 0.40*^a^*
X1-BX4 (aa 133–184)	4561 ± 499*^a^*	4.28 ± 0.04*^b^*	59.5 ± 2.9*^b^*	5.57 ± 0.09*^a^*	1.26 ± 0.11*^a^*	5.24 ± 0.12*^d^*	1.72 ± 0.32*^a^*	7.28 ± 0.09*^b^*	1.09 ± 0.22*^a^*
X1-CX4 (aa 185–261)	8546 ± 747*^a^*	6.17 ± 0.13*^a^*	25.3 ± 2.9*^a^*	5.61 ± 0.13*^a^*	0.84 ± 0.11*^a^*	5.47 ± 0.12*^a^*	0.79 ± 0.12*^a^*	6.46 ± 0.19*^b^*	1.05 ± 0.42*^a^*
X1-DX4 (aa 262–330)	5265 ± 546*^a^*	5.08 ± 0.04*^b^*	40.1 ± 2.1*^c^*	6.75 ± 0.08*^b^*	1.10 ± 0.11*^a^*	6.95 ± 0.07*^b^*	1.72 ± 0.23*^a^*	8.42 ± 0.08*^d^*	1.51 ± 0.31*^a^*

*^a^* Not significant.

*^b^* Significant difference from the hP2X1 receptor (*p* < 0.0001).

*^c^* Significant difference from the hP2X1 receptor (*p* < 0.01).

*^d^* Significant difference from the hP2X1 receptor (*p* < 0.05).

##### Antagonist Action at hP2X1 Receptor Chimeras

**FIGURE 2.**
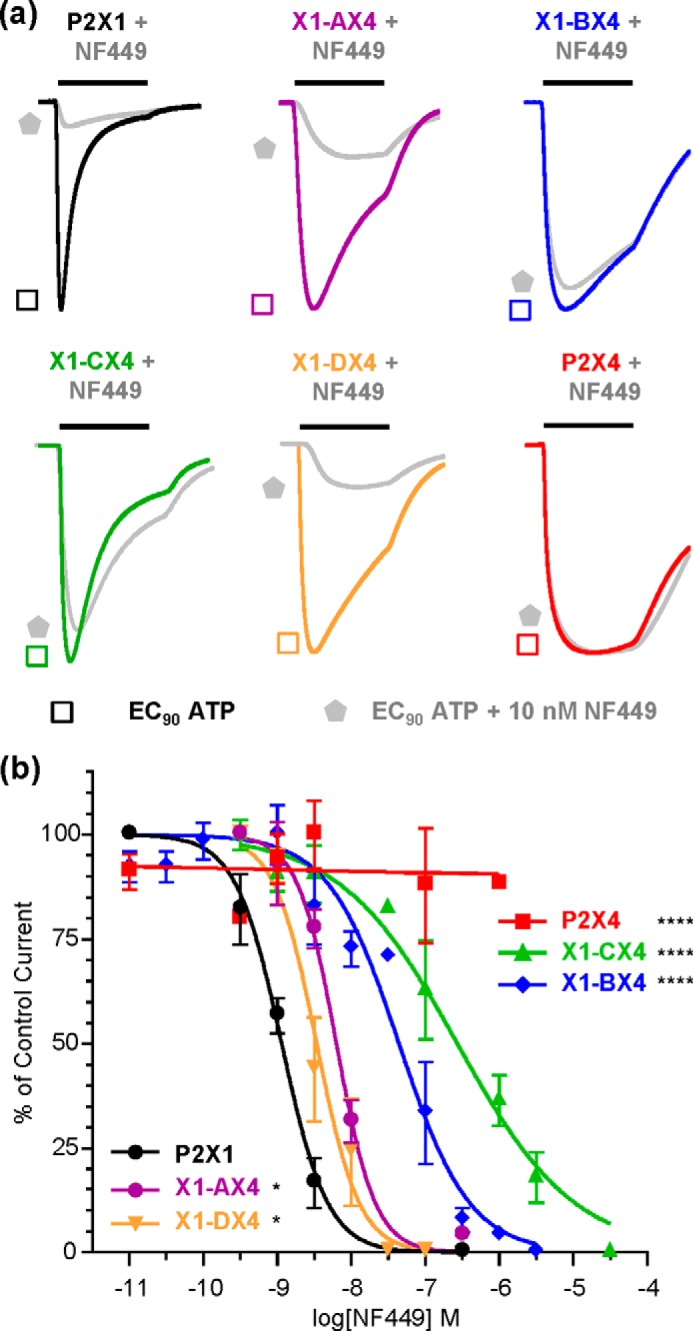
**NF449 action at chimeric P2X receptors.** *a*, representative traces showing the effect of NF449 (10 nm) on currents evoked by an EC_90_ of ATP in *Xenopus* oocytes expressing WT and chimeric receptors. The *bars* indicate a 3-s agonist/antagonist application. *b*, NF449 inhibition curves at an EC_90_ of ATP. *Asterisks* indicate a significant shift in IC_50_ from the hP2X1 receptor. *, *p* < 0.05; ****, *p* < 0.0001.

A standardized EC_90_ concentration of ATP at each receptor was used to characterize antagonist sensitivity. NF449 inhibited ATP responses with an IC_50_ of ∼1 nm at the hP2X1 receptor and even at 1 μm had no effect at the rP2X4 receptor, consistent with previous reports (15, 21, 22). The chimeras showed decreased NF449 sensitivity compared with the hP2X1 receptor (Fig. 2). This was <5-fold for X1-AX4 and X1-DX4, ∼60-fold for X1-BX4, and ∼135-fold for X1-CX4 (Fig. 2 and Table 1).

**FIGURE 3.**
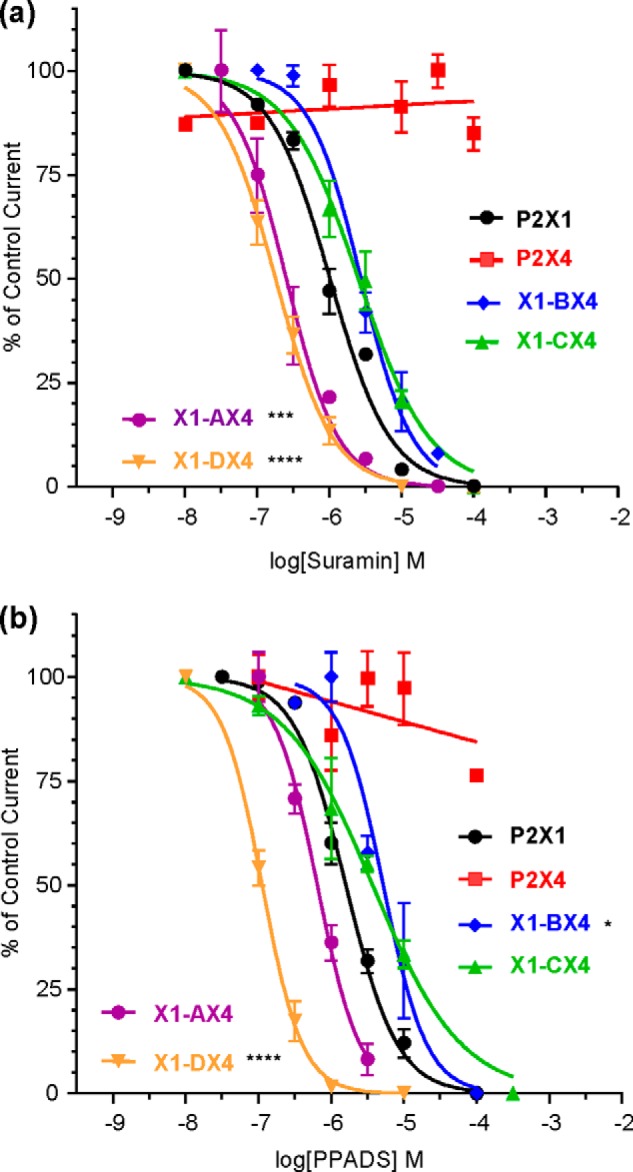
**Suramin and PPADS antagonism at chimeric P2X receptors.** *a*, comparison of suramin action in WT receptors and chimeras. *b*, PPADS inhibition curves for chimeric and WT receptors. In all cases, antagonist action was determined against an EC_90_ of ATP. *Asterisks* indicate a significant shift from the WT P2X1 receptor. **, *p* < 0.05; ***, *p* < 0.0001.

The antagonists suramin and PPADS inhibited the hP2X1 receptor (IC_50_ ∼ 1 μm) but were ineffective (100 μm) in the rP2X4 receptor, as reported previously (15, 20, 21, 29). Sensitivity to suramin or PPADS in the X1-CX4 chimera was the same as that in the hP2X1 receptor (Fig. 3 and Table 1). Paradoxically, sensitivity to both suramin and PPADS was actually increased by ∼10-fold for the X1-DX4 chimera compared with the hP2X1 receptor (*p* < 0.0001) (Fig. 3 and Table 1). The remaining chimeras showed either an ∼4-fold increase for suramin (*p* < 0.001) with no effect on PPADS (X1-AX4) or an ∼3.5-fold decrease in PPADS inhibition (*p* < 0.05) with no effect on suramin sensitivity (X1-BX4) (Fig. 3 and Table 1). The predominant finding of a lack of decrease in suramin or PPADS action at the chimeras contrasts with the decrease in NF449 sensitivity. This suggests that antagonist insensitivity at the rP2X4 receptor has multiple underlying molecular mechanisms dependent on the antagonist. The large decreases in NF449 sensitivity for chimeras X1-BX4 and X1-CX4 suggests that variant residues in these regions are likely to make an important contribution to NF449 action and were thus the focus of further studies.

##### Decreased NF449 Sensitivity at the X1-BX4 Chimera Is Due to Removal of Positive Charge at the Base of the Cysteine-rich Head Region

**FIGURE 4.**
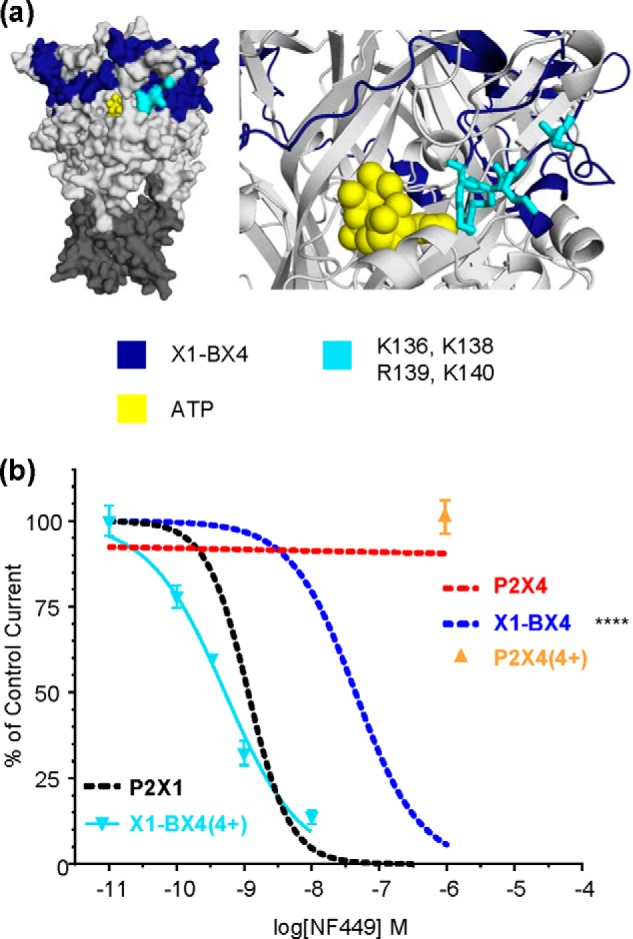
**Introduction of four charges reintroduced NF449 sensitivity to the X1-BX4 chimera.** *a*, location of Lys-136, Lys-138, Arg-139, and Lys-140 in a P2X1 receptor homology model. Docked ATP is shown in *yellow. b*, NF449 inhibition curves showing the effect of reintroducing the positive charges to the X1-BX4 chimera and NF449 action in the P2X4(4+) receptor. *Asterisks* indicate a significant difference in IC_50_ compared with the P2X1 receptor. ****, *p* < 0.0001.

The ∼60-fold decrease in NF449 sensitivity at the X1-BX4 chimera was consistent with our previous study showing that mutating four positive charges at the base of the cysteine-rich head region reduced sensitivity to the antagonist (19). However, as well as the four positive charges, the region contained an additional 20 residues that were variant. To determine whether it was solely the replacement of the positive charges that was responsible for the decrease in NF449 sensitivity of X1-BX4 or whether other residues within region B were involved, the positive charges were mutated back into the X1-BX4 chimera (Fig. 4). The mutant chimera incorporating the mutations to reintroduce the charges (S136K, D138K, T139R, and H140K; named X1-BX4(4+)) reduced ATP potency further (∼85-fold from hP2X1 receptors and ∼10-fold from X1-BX4; pEC_50_ = 3.6 ± 0.1; *p* < 0.0001 and 0.05 for P2X1 and X1-BX4, respectively) but interestingly returned the level of desensitization (15.7 ± 1.7% current remaining at the end of application) to hP2X1 receptor levels. These results show that the positively charged residues present in the cysteine-rich head region of the WT hP2X1 receptor contribute to its characteristic fast time course and that this is independent of effects on ATP sensitivity. The NF449 sensitivity of the X1-BX4(4+) mutant was returned to hP2X1 receptor levels (pIC_50_ = 9.28 ± 0.05 and 8.94 ± 0.04, respectively). This demonstrates the importance of the four positive charges at the base of the cysteine-rich head region in antagonist action. In addition, it suggests that the other 20 variant residues between hP2X1 and rP2X4 receptors in region B are unlikely to account for the insensitivity to NF449 in the rP2X4 receptor.

As the presence of four positive charges reintroduced nanomolar NF449 sensitivity to the X1-BX4 chimera, we hypothesized that their introduction to the rP2X4 receptor could make the receptor NF449-sensitive. The positive charges were therefore introduced at the equivalent positions of the rP2X4 receptor (S136K, D138K, T139R, and H140K) to make the P2X4(4+) mutant. When 1 μm NF449 was co-applied with the EC_90_ of ATP to the P2X4(4+) receptor, no inhibition of the peak current was seen (103.5 ± 5.3% of control). This shows that the presence of the four charges alone is not enough to cause the rP2X4 receptor to be inhibited by NF449. This is consistent with previous studies on the hP2X2 receptor, where introduction of the four positive charges had no effect on NF449 sensitivity (19), and indicates that a combination of residues are required for high affinity NF449 binding.

##### Determination of Role of Variant Regions in the X1-CX4 Chimera to NF449 Action

**FIGURE 5.**
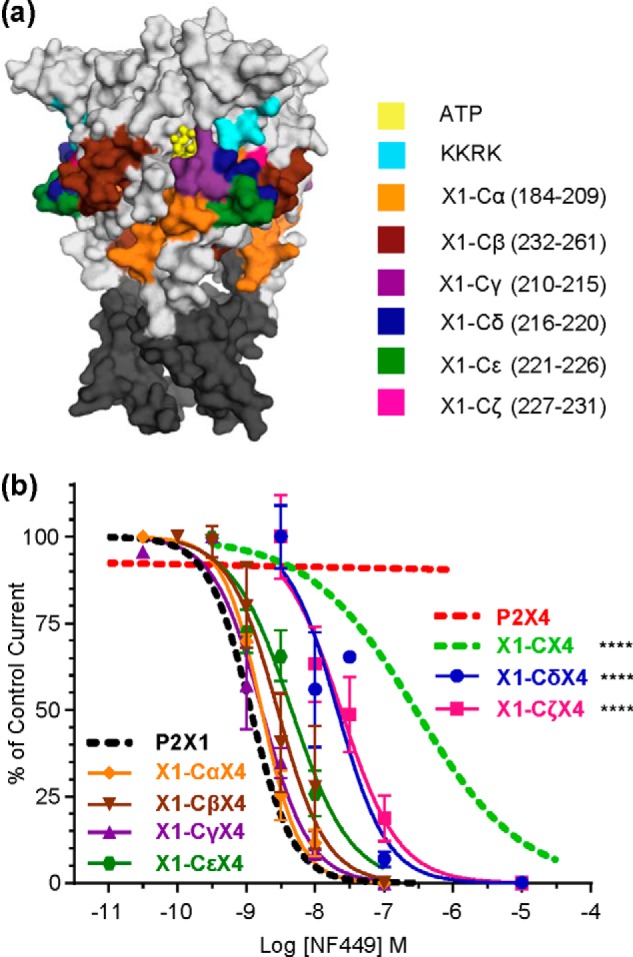
**Effects of NF449 at region C subchimeras.** *a*, location of residues swapped to generate chimeras shown in a homology model of the P2X1 receptor. Docked ATP is shown in *yellow*, and transmembrane domains are shown in *dark gray. b*, NF449 inhibition curves for subchimeras. Curves were generated at an EC_90_ of ATP. *Asterisks* represent a significant difference in IC_50_ compared with the P2X1 receptor. ****, *p* < 0.0001.

The ∼135-fold decrease in NF449 sensitivity at the X1-CX4 chimera indicates that the variant residues in region C (residues 185–261) contribute to antagonist action. The region swapped in chimera X1-CX4 is located below the cysteine-rich head region. Given the importance of positive charge in the cysteine-rich head region to NF449 sensitivity, we reasoned that residues in the X1-CX4 chimera also important to NF449 sensitivity would be nearby. To test this, we split region C into two larger subchimeras (X1-CαX4 (residues 184–209) and X1-CβX4 (residues 232–261)) that were most distant from the head region and four smaller chimeras in closer proximity **(**X1-CγX4 (residues 210–215), X1-CδX4 (residues 216–220), X1-CϵX4 (residues 221–226), and X1-CζX4 (residues 227–231)) (Fig. 5). Chimeras X1-CγX4 and X1-CϵX4 had hP2X1 receptor ATP sensitivity. In contrast, it was significantly reduced (*p* < 0.0001) for the remaining chimeras, and in the case of X1-CδX4 and X1-CζX4, it was reduced to rP2X4 receptor levels (pEC_50_ = 5.21 ± 0.04 and 5.54 ± 0.06, respectively) (Table 2). For all region C subchimeras, the desensitization was equivalent to hP2X1 receptor levels (10–35% of peak current remaining at 3 s).

**TABLE 2:** **ATP and NF449 properties of region C subchimeras** NF449 properties were determined at the EC_90_ of ATP. aa, amino acids; ND, not determined.

	Peak current	ATP			NF449	
		pEC_50_	Current remaining at 3 s	Hill slope	pIC_50_	Hill slope
	*nA*		%			
P2X1	7598 ± 793	6.31 ± 0.04	19.7 ± 4.3	0.89 ± 0.07	8.94 ± 0.04	1.40 ± 0.20
P2X4	5320 ± 586*^a^*	5.39 ± 0.10*^b^*	71.4 ± 4.5*^b^*	1.07 ± 0.12*^a^*	≪6.00	ND
X1-CX4 (aa 185–261)	8546 ± 747*^a^*	6.17 ± 0.13*^a^*	25.3 ± 2.9*^a^*	0.67 ± 0.06*^a^*	6.46 ± 0.19*^b^*	1.05 ± 0.42*^a^*
X1-CαX4 (aa 185–209)	6893 ± 864*^a^*	5.34 ± 0.05*^b^*	10.6 ± 1.5*^a^*	0.97 ± 0.11*^a^*	8.78 ± 0.04*^a^*	1.56 ± 0.22*^a^*
X1-CβX4 (aa 232–261)	6117 ± 669*^a^*	5.30 ± 0.06*^b^*	15.2 ± 8.2*^a^*	1.06 ± 0.14*^a^*	8.58 ± 0.09*^a^*	1.13 ± 0.09*^a^*
X1-CγX4 (aa 210–215)	7623 ± 767*^a^*	5.71 ± 0.06*^a^*	34.1 ± 2.3*^a^*	1.13 ± 0.06*^a^*	8.78 ± 0.06*^a^*	1.25 ± 0.22*^a^*
X1-CδX4 (aa 216–220)	8560 ± 800*^a^*	5.21 ± 0.04*^b^*	24.7 ± 2.3*^a^*	1.49 ± 0.18*^a^*	7.68 ± 0.12*^b^*	1.20 ± 0.32*^a^*
X1-CϵX4 (aa 221–226)	7047 ± 588*^a^*	5.68 ± 0.06*^a^*	23.5 ± 3.7*^a^*	1.20 ± 0.19*^a^*	8.36 ± 0.09*^a^*	1.16 ± 0.30*^a^*
X1-CζX4 (aa 227–231)	7450 ± 639*^a^*	5.54 ± 0.06*^b^*	19.0 ± 8.2*^a^*	0.94 ± 0.11*^a^*	7.63 ± 0.11*^b^*	1.04 ± 0.26*^a^*

*^a^* Not significant.

*^b^* Significant difference from the hP2X1 receptor (*p* < 0.0001).

NF449 sensitivity was equivalent to hP2X1 receptor levels in the X1-CαX4, X1-CβX4, X1-CγX4, and X1-CϵX4 chimeras and was reduced by ∼20-fold for X1-CδX4 and X1-CζX4 (*p* < 0.0001) (Fig. 5 and Table 2). No one chimera replicated the decrease in sensitivity to NF449 of the X1-CX4 chimera. This suggests that a combination of residues within subchimeras of region C, closest to the four positively charged residues in the cysteine-rich head region, contribute to NF449 sensitivity.

##### Importance of Individual Variant Residues in Region C Chimeras to NF449 Action

**FIGURE 6.**
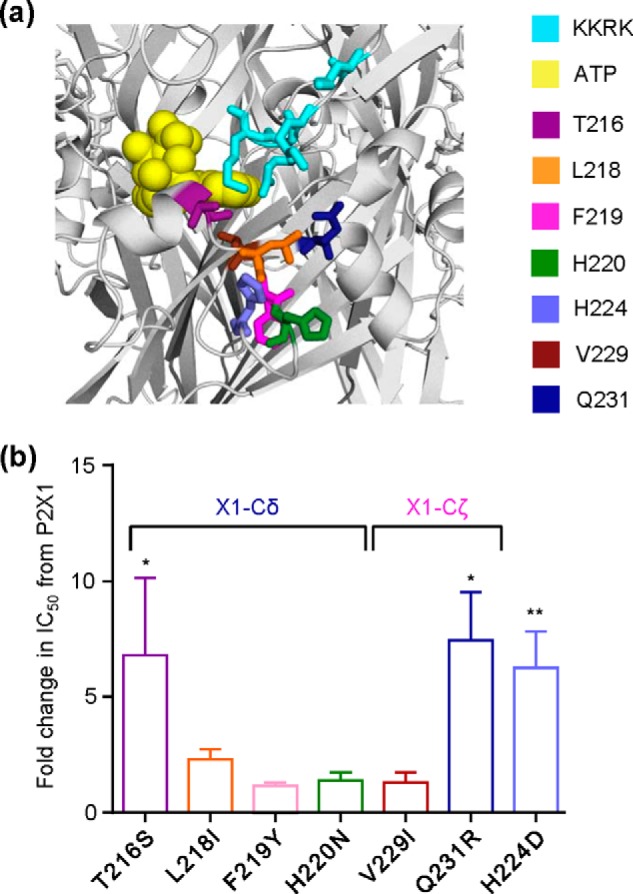
**Effects of NF449 at point mutants.** *a*, location of point mutated residues in a P2X1 receptor homology model. Docked ATP is shown in *yellow*. Residues were mutated to the equivalent residue of the P2X4 receptor. *b*, histogram showing the -fold change in NF449 inhibition compared with the WT P2X1 receptor at an EC_90_ of ATP. *Asterisks* represent a significant difference. *, *p* < 0.05; **, *p* < 0.01.

For the subchimeras with a decrease in NF449 sensitivity (X1-CδX4 and X1-CζX4), individual point mutations of rP2X4 receptor variant residues within these regions were introduced to the hP2X1 receptor. The mutations made were T216S, L218I, F219Y, H220N, V229I, and Q231R (Fig. 6). All of the mutants were functional and showed hP2X1 receptor ATP sensitivity and levels of desensitization with the exception of H220N, which had an ∼8-fold decrease in ATP sensitivity (*p* < 0.001) and reduced desensitization (54.2 ± 5.1% current remaining at the end of agonist application compared with 19.7 ± 4.3% for hP2X1 receptors). There was no change in NF449 inhibition compared with the hP2X1 receptor for the L218I, F219Y, H220N, and V229I mutants (Fig. 6). The mutation at position 216, which mutated a threonine residue to a serine, decreased NF449 sensitivity by ∼7-fold (*p* < 0.05) (Fig. 6). The Q231R mutation, which introduced a positively charged residue beneath the four charges in the cysteine-rich head region, also showed a decrease in NF449 sensitivity of ∼7-fold (*p* < 0.01) (Fig. 6). These results highlight individual residues below the cysteine-rich head region that contribute to NF449 sensitivity.

##### Molecular Modeling of NF449 Binding and Validation by Site-directed Mutagenesis

**FIGURE 7.**
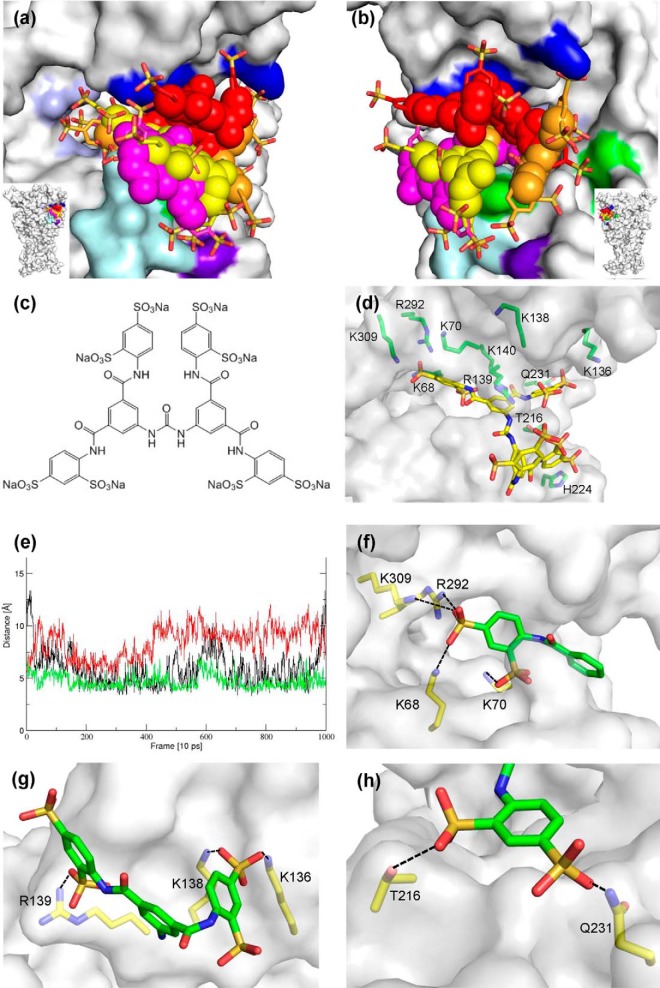
**Docking poses for the NF449-P2X1 complex.** *a* and *b*, overlay of the four docking poses A–D on the P2X1 receptor shown from different angles. The P2X1 receptor is shown in surface representation, highlighting the positively charged residues of the ATP-binding site (Lys-68, Lys-70, Arg-292, and Lys-309) in *light blue*, the positively charged residues of the cysteine-rich head region (Lys-136, Lys-138, Arg-139, and Lys-140) in *dark blue*, Thr-216 and Gln-231 in *green*, region X1-CγX4 (residues 210–215; see “Results”) in *pale cyan*, and His-224 in *purple*. NF449 poses A (*red*), B (*orange*), C (*pink*), and D (*yellow*) are shown as a mixture of sphere representation (for the core of the poses) and stick representation (for the arms of the poses). All four poses bind to the cleft between the cysteine-rich head region, ATP-binding site, and dorsal fin. *c*, molecular structure of the NF449 sodium salt. *d*, snapshot of NF449 from the pose C trajectory. NF449 and residues Lys-68, Lys-70, Lys-136, Lys-138, Arg-139, Lys-140, Thr-216, His-224, Gln-231, Arg-292, and Lys-309 are shown in stick representation. *e*, distances between NF449 sulfonate sulfur atoms and His-224 Nϵ (*black*), Thr-216 Oγ (*green*), and the amide nitrogen of Gln-231 monitored over the 10-ns molecular dynamics simulation of pose C. NF449/His-224 and NF449/Thr-216 interactions are present in most of the frames, and NF449/Gln-231 interactions are less stable in this simulation and are present only between 1.5 and 4.5 ns. *f*, section of NF449 pose C interacting with the ATP-binding site. Potential salt bridges are indicated by *dashed black lines. g*, section of NF449 pose A and residues Lys-136, Lys-138, and Arg-139 of the cysteine-rich head region. *h*, arm of NF449 pose D forming H-bonds with Thr-216 and Gln-231 side chains.

The six residues (Lys-136, Lys-138, Arg-139, Lys-140, Thr-216, and Gln-231) that were identified in the first round of site-directed mutagenesis as contributing to inhibition of the hP2X1 receptor by NF449 were used to define the overall docking site on the hP2X1 apo form homology models, but no specific constraints were enforced. Ligand docking provides a series of alternative poses ranked by docking scores. For the interpretation of models, considering agreement with experimental data in addition to docking scores is paramount. All NF449 docking poses with Astex statistical potential scores of >35 were visualized and filtered manually for their interactions with the six residues contributing to NF449 antagonism. The four poses matching both criteria have some key interactions with NF449 in common (Table 3). A characteristic feature of NF449 could be described as its four phenyl “arms,” each with two sulfonic acid groups in *ortho*- and *para*-positions (*ortho* and *para* defined in relation to the amide linker). These negatively charged arms would be expected to form salt bridges or hydrogen bonds with the receptor. All four docking poses show one arm forming salt bridges with at least two of the positively charged residues of the ATP-binding site (Lys-68, Lys-70, Arg-292, and Lys-309). This suggests that NF449 partially occupies the ATP-binding pocket, accounting for its competitive antagonist characteristics. However, the majority of NF449 interactions with P2X1 take place outside of the ATP-binding pocket (as predicted due to the larger size of NF449). In all four poses, a second arm is involved in salt bridges with the block of positively charged residues at the base of the cysteine-rich head region (Lys-136, Lys-138, Arg-139, and Lys-140), although the specifics of these interactions vary between the four poses (Fig. 7). All four poses occupy the cavity under the cysteine-rich head region close to site of ATP binding, which could prevent the movement of the cysteine-rich head region upon ATP binding (9). In poses A, C, and D (and to a lesser extent, pose B), a third arm of NF449 interacts with the side chains of Thr-216 and Gln-231. It is also noteworthy that the conserved residue Lys-215 contributes to salt bridges in all four poses.

**TABLE 3:** **Salt bridge and hydrogen bond interactions of the four docking poses A–D with key residues of the P2X1 receptor model** *ortho* and *para* refer to the sulfonate groups in the *ortho*- and *para*-positions relative to the amide linker.

Residue	Pose A	Pose B	Pose C	Pose D
Lys-136	*para*	*para*		
Lys-138	*para*			
Arg-139	*ortho*	*ortho*	*ortho/para*	*ortho/ortho*
Lys-140		*ortho*	*para*	*para*
Lys-68	*para*		*para*	
Lys-70	*ortho*	*ortho*	*ortho*	
Arg-292			*para*	*para*
Lys-309		*para*	*para*	*para*
Thr-216	*ortho*		*ortho*	*ortho*
Gln-231	*para*		*para*	*para*
His-224		*para*	*para*	*para*
Lys-215	*para*	*ortho*	*ortho*	*ortho*

Although the four poses have key features in common, there are also differences. For example, poses B–D predict an interaction of one NF449 arm with His-224, an interaction not present in pose A. Interestingly, His-224 is part of the X1-CϵX4 chimera, with a 4-fold decrease in NF449 sensitivity. To test whether this predicted interaction could be confirmed, we generated the H224D mutant (of the rP2X4 receptor residue). The H224D mutation (with no effect on ATP sensitivity or time course), which introduced a negatively charged residue beneath the four positively charged residues in the cysteine-rich head region, produced an ∼6-fold decrease (*p* < 0.05) in NF449 sensitivity compared with the hP2X1 receptor (Fig. 6). This supports the idea that this residue is indeed involved in NF449 binding. Taken together with the data above, specifically the lack of favorable interactions between pose B and residues Thr-216 and Gln-231, poses C and D are in better agreement with the experimental data than poses A and B.

##### Analysis of NF449 Binding with Molecular Dynamics Simulations

Molecular dynamics simulations are an efficient way of refining structural models, testing their stability, and improving conformational sampling. To further analyze and refine the four predicted poses from ligand docking in an unbiased way, four 10-ns molecular dynamics simulations were run. The molecular dynamics trajectories of the extracellular domains of the P2X1 models with NF449 in poses A–D were analyzed to test whether the interactions found by ligand docking are stable over the time course of the simulations.

All four poses maintained favorable interactions between NF449 and positively charged residues of the cysteine-rich region (Lys-136, Lys-138, Arg-139, and Lys-140) and between NF449 and positively charged residues within the ATP-binding site (Lys-68, Lys-70, Arg-292, and Lys-309), but differences were found for interactions with Thr-216, His-224, and Gln-231. For pose A, His-224 Nδ and His-224 Nϵ are found within distances of 8–12 Å from the closest NF449 sulfonate group over the full time course of the simulation, too far for a strong charge interaction. The trajectory of pose B shows a stable interaction between an NF449 sulfonate group and His-224 over the entire simulation, but favorable interactions of NF449 and residues Thr-216 and Gln-231 are present in only <10% of the trajectory time points. Docking poses C and D occupy roughly the same space between the cysteine-rich head and the ATP-binding site. Over the time course of the simulations, pose C maintained the interactions between NF449 sulfonate groups and Thr-216 and His-224 and, to an extent, Gln-231 (Fig. 7*e*). This was not the case for pose D; here, none of these interactions were maintained. Hence, structural models derived from the trajectory of pose C are in best agreement with the experimental data.

## DISCUSSION

P2X receptors share a conserved core ATP-binding site that forms at the interface between adjacent subunits (9). Variations in amino acid sequence in the rest of the receptor give rise to a range of subtype-dependent properties. The chimeras between the hP2X1 and rP2X4 receptors in this study have been useful to identify whether a region contributes to differences in properties, *e.g.* ATP sensitivity and desensitization. Replacing residues 185–261 (chimera X1-C4) of the hP2X1 receptor with those of the rP2X4 receptor had no effect on ATP potency or desensitization, demonstrating that variations in this region (below the ATP-binding pocket) do not contribute to the differences in these properties. In contrast, ATP sensitivity and desensitization was decreased (compared with hP2X1 receptors) in chimeras X1-BX4 and X1-DX4. It seems unlikely that these changes result from gross conformational effects on the receptor, as chimera X1-BX4, which showed the largest decrease in ATP potency (∼100-fold less than hP2X1 and ∼10-fold less than rP2X4), had no effect on inhibition by suramin. These results suggest that variations in regions B and D (residues 133–184 and 262–330) contribute to the differences in ATP responses. They also raise the possibility that regulation of agonist potency and time course are interdependent. However, this is not the case, as the X1-AX4 chimera had an ∼100-fold decrease in ATP potency with no effect on desensitization. This supports studies showing that agonist sensitivity and time course can be regulated independently (25, 30). Our findings complement work showing the importance of residues throughout the intracellular, transmembrane, and extracellular regions in the regulation of ATP responsiveness (4) and highlight that modifications throughout the whole receptor can regulate agonist binding and channel gating.

The >1000-fold difference in NF449, suramin, and PPADS inhibition between the hP2X1 (sensitive) and rP2X4 (insensitive) receptors was the starting point for this study. The ≥60-fold decrease in NF449 antagonism at both X1-BX4 and X1-CX4 mutants showed that a chimeric approach could identify variant regions contributing to antagonist action. It was therefore surprising that none of the chimeras showed a reduction in suramin sensitivity, and the affinity was actually increased by ∼10-fold for the X1-AX4 and X1-DX4 chimeras. A similar result was seen for the actions of PPADS, with only a modest 3-fold decrease in sensitivity for X1-BX4, no change for X1-AX4 and X1-CX4, and an ∼10-fold increase in affinity for X1-DX4. These results show that sections of the suramin- and PPADS-insensitive rP2X4 receptor can substitute for those of the hP2X1 receptor, and a significant number of residues required for suramin and PPADS action are already present in the rP2X4 receptor. This is consistent with studies showing that point mutations or changing small sections increased suramin and PPADS sensitivity at P2X4 receptors (15, 16, 31). For example, PPADS sensitivity can be increased in the rP2X4 receptor with the mutation of the negatively charged aspartate residue at position 249 to the positive lysine residue found at the equivalent position in the PPADS-sensitive P2X1 and P2X2 receptors (15). However, the reciprocal mutation in the P2X2 receptor did not reduce the level of PPADS inhibition (15). This suggests that the contribution of a particular residue is dependent on the receptor background, *i.e.* a number of factors combine to regulate inhibition. Whether this corresponds to the complement of residues in the antagonist-binding site(s) and/or the conformation(s) the receptor adopts remains to be determined.

The ∼60-fold decrease in NF449 antagonism at the X1-BX4 chimera could be attributed to the replacement of four positive charges at the base of the cysteine-rich loop, as sensitivity was completely rescued at the X1-BX4(4+) mutant chimera. This is consistent with previous studies showing the importance of positive charge at this position in NF449 action (18, 19). In addition, the rescue of NF449 sensitivity at the X1-BX4(4+) chimera indicates that the other 20 variant residues in the BX4 segment are unlikely to contribute to sensitivity of the antagonist (consistent with ligand docking studies).

The action of ATP at the X1-CX4 chimera was identical to that at the hP2X1 receptor; however, the sensitivity to NF449 was reduced by ∼135-fold. Subdivision of the chimera identified the dorsal fin region (14) below the positive charges of the cysteine-rich head as contributing to the decrease in NF449 action. Within this region, the point mutations (hP2X1-rP2X4) T216S and Q231R reduced NF449 sensitivity by ∼7-fold, indicating that these variations contribute to the reduction in antagonist affinity. Interestingly, in all other mammalian P2X receptor subunits (except P2X5), the residue corresponding to Gln-231 is a positive charge, suggesting that this variation contributes in part to the selectivity of NF449 at P2X1 receptors. In addition, there is some modest difference in NF449 sensitivity between species orthologs of the P2X1 receptor (18, 32) that may result in part from variation in the residue at position 231 (human, Gln; rat, Asn; mouse, Ser) and/or 216 (human, Thr; rat and mouse, Lys). Molecular modeling suggests a mechanistic explanation for the effect of the T216S and Q231R mutations. In docking poses B–D, one of the benzenedisulfonic acid arms of NF449 is anchored between the side chains of Thr-216 and Gln-231 so that Thr-216 forms an H-bond with the sulfonic acid group in the *ortho*-position, whereas Gln-231 interacts with the sulfonic acid group in the *para*-position (Fig. 7*d*). Such an arrangement may be sterically less favorable for the T216S and Q231R mutants. Interestingly, the NF449 analogs NF110 (sulfonic acid only in the *para*-position) and MK3 (sulfonic acid only in the *ortho*-position) show significantly lower *K_i_* than NF449 (33). The bidental arrangement of Thr-216 and Gln-231 seems ideal for binding NF449, but less so for its analogs with only one substituent. This may provide a rational for the observation that the *K_i_* of NF449-P2X1 is ∼800-fold lower than that of NF110-P2X1 (250-fold lower for MK3-P2X1), in contrast to rP2X2 and rP2X3, where the *K_i_* of NF449 is actually higher compared with its NF110 and MK3 analogs (33).

The identification of positive charges at the base of the cysteine-rich head region and residues Thr-216 and Gln-231 as being important in NF449 action was the starting point for molecular docking studies. These identified four clusters of solutions around the cysteine-rich head region, with part of the NF449 molecule being coordinated by residues within the core ATP-binding pocket conserved between P2X receptor subunits. A common feature of the models was the binding of part of NF449 within the agonist pocket. This is consistent with the antagonist action of NF449 at non-P2X1 receptor subunits, albeit with >1000-fold reduced affinity at the P2X2 (19, 22), P2X3 (34), P2X4 (22), and P2X7 (32) receptors. Direct overlap of part of the NF449-binding site would also account for the competitive nature of the antagonism (32).

Our work is a major step forward in understanding the specificity of NF449 antagonist action at the hP2X1 receptor. The combination of chimeras, point mutants, and molecular modeling suggests that the interplay of four key regions of interactions is of importance for NF449 binding to P2X1 receptors (Fig. 7). The interaction of NF449 with positively charged residues of the ATP-binding site cannot explain NF449 specificity, as these residues are essential for ATP binding and are conserved across other P2X receptors. However, the positively charged residues Lys-136, Lys-138, Arg-139, and Lys-140 within the cysteine-rich head region are exclusively found at P2X1 receptors. Similarly, Thr-216 and Gln-231 are not conserved across the P2X receptor family, and His-224 corresponds to an aspartic acid at P2X2, P3X3, and P2X4. We have shown that these sites affect NF449 potency. As they are particular to hP2X1 receptors and are not present in other P2X receptors, we conclude that their interactions with NF449 are likely to be key to NF449 specificity and that they are of significant interest for the development of P2X1 receptor-specific drugs.

## Footnotes

2 The abbreviations used are:

NF449: 4,4′,4″,4‴-(carbonylbis(imino-5,1,3-benzenetriylbis(carbonylimino)))tetrakisbenzene-1,3-disulfonic acid
PPADS: pyridoxal-phosphate-6-azophenyl-2,4-disulfonate
hP2X1: human P2X1
rP2X4: rat P2X4.

## References

[1] Burnstock G. (2012) Purinergic signalling: its unpopular beginning, its acceptance and its exciting future. BioEssays 34, 218–225 22237698 10.1002/bies.201100130

[2] Valera S. Hussy N. Evans R. J. Adami N. North R. A. Surprenant A. Buell G. (1994) A new class of ligand-gated ion channel defined by P2X receptor for extracellular ATP. Nature 371, 516–519 7523951 10.1038/371516a0

[3] Brake A. J. Wagenbach M. J. Julius D. (1994) New structural motif for ligand-gated ion channels defined by an ionotropic ATP receptor. Nature 371, 519–523 7523952 10.1038/371519a0

[4] Kaczmarek-Hájek K. Lörinczi E. Hausmann R. Nicke A. (2012) Molecular and functional properties of P2X receptors–recent progress and persisting challenges. Purinergic Signal. 8, 375–417 22547202 10.1007/s11302-012-9314-7 PMC3360091

[5] Surprenant A. North R. A. (2009) Signaling at purinergic P2X receptors. Annu. Rev. Physiol. 71, 333–359 18851707 10.1146/annurev.physiol.70.113006.100630

[6] Hechler B. Lenain N. Marchese P. Vial C. Heim V. Freund M. Cazenave J.-P. Cattaneo M. Ruggeri Z. M. Evans R. Gachet C. (2003) A role of the fast ATP-gated P2X_1_ cation channel in the thrombosis of small arteries *in vivo*. J. Exp. Med. 198, 661–667 12913094 10.1084/jem.20030144 PMC2194166

[7] Oury C. Kuijpers M. J. Toth-Zsamboki E. Bonnefoy A. Danloy S. Vreys I. Feijge M. A. De Vos R. Vermylen J. Heemskerk J. W. Hoylaerts M. F. (2003) Overexpression of the platelet P2X_1_ ion channel in transgenic mice generates a novel prothrombotic phenotype. Blood 101, 3969–3976 12521992 10.1182/blood-2002-10-3215

[8] Hechler B. Magnenat S. Zighetti M. L. Kassack M. U. Ullmann H. Cazenave J.-P. Evans R. Cattaneo M. Gachet C. (2005) Inhibition of platelet functions and thrombosis through selective or nonselective inhibition of the platelet P2 receptors with increasing doses of NF449 [4,4′,4″,4‴[-(carbonylbis(imino-5,1,3-benzenetriylbis-(carbonylimino)))tetrakis-benzene-1,3-disulfonic acid octasodium salt]. J. Pharmacol. Exp. Ther. 314, 232–243 15792995 10.1124/jpet.105.084673

[9] Hattori M. Gouaux E. (2012) Molecular mechanism of ATP binding and ion channel activation in P2X receptors. Nature 485, 207–212 22535247 10.1038/nature11010 PMC3391165

[10] Evans R. J. (2010) Structural interpretation of P2X receptor mutagenesis studies on drug action. Br. J. Pharmacol. 161, 961–971 20977449 10.1111/j.1476-5381.2010.00728.x PMC2972645

[11] Browne L. E. Jiang L. H. North R. A. (2010) New structure enlivens interest in P2X receptors. Trends Pharmacol. Sci. 31, 229–237 20227116 10.1016/j.tips.2010.02.004 PMC2954318

[12] Young M. T. (2010) P2X receptors: dawn of the post-structure era. Trends Biochem. Sci. 35, 83–90 19836961 10.1016/j.tibs.2009.09.006 PMC2824114

[13] Chataigneau T. Lemoine D. Grutter T. (2013) Exploring the ATP-binding site of P2X receptors. Front. Cell. Neurosci. 7, 273 24415999 10.3389/fncel.2013.00273 PMC3874471

[14] Kawate T. Michel J. C. Birdsong W. T. Gouaux E. (2009) Crystal structure of the ATP-gated P2X_4_ ion channel in the closed state. Nature 460, 592–598 19641588 10.1038/nature08198 PMC2720809

[15] Buell G. Lewis C. Collo G. North R. A. Surprenant A. (1996) An antagonist-insensitive P2X receptor expressed in epithelia and brain. EMBO J. 15, 55–62 8598206 PMC449917

[16] Garcia-Guzman M. Soto F. Gomez-Hernandez J. M. Lund P.-E. Stühmer W. (1997) Characterization of recombinant human P2X_4_ receptor reveals pharmacological differences with the rat homologue. Mol. Pharmacol. 51, 109–118 9016352 10.1124/mol.51.1.109

[17] Jarvis M. F. Khakh B. S. (2009) ATP-gated P2X cation-channels. Neuropharmacology 56, 208–215 18657557 10.1016/j.neuropharm.2008.06.067

[18] Sim J. A. Broomhead H. E. North R. A. (2008) Ectodomain lysines and suramin block of P2X_1_ receptors. J. Biol. Chem. 283, 29841–29846 18765669 10.1074/jbc.M802523200 PMC2573084

[19] El-Ajouz S. Ray D. Allsopp R. C. Evans R. J. (2012) Molecular basis of selective antagonism of the P2X1 receptor for ATP by NF449 and suramin; contribution of basic amino acids in the cysteine-rich loop. Br. J. Pharmacol. 165, 390–400 21671897 10.1111/j.1476-5381.2011.01534.x PMC3268193

[20] Soto F. Garcia-Guzman M. Gomez-Hernandez J. M. Hollmann M. Karschin C. Stühmer W. (1996) P2X_4_: an ATP-activated ionotropic receptor cloned from rat brain. Proc. Natl. Acad. Sci. U.S.A. 93, 3684–3688 8622997 10.1073/pnas.93.8.3684 PMC39672

[21] Bo X. Zhang Y. Nassar M. Burnstock G. Schoepfer R. (1995) A P2X purinoceptor cDNA conferring a novel pharmacological profile. FEBS Lett. 375, 129–133 7498461 10.1016/0014-5793(95)01203-q

[22] Rettinger J. Braun K. Hochmann H. Kassack M. U. Ullmann H. Nickel P. Schmalzing G. Lambrecht G. (2005) Profiling at recombinant homomeric and heteromeric rat P2X receptors identifies the suramin analogue NF449 as a highly potent P2X_1_ receptor antagonist. Neuropharmacology 48, 461–468 15721178 10.1016/j.neuropharm.2004.11.003

[23] Ennion S. Hagan S. Evans R. J. (2000) The role of positively charged amino acids in ATP recognition by human P2X_1_ receptors. J. Biol. Chem. 275, 29361–29367 10827197 10.1074/jbc.M003637200

[24] Royle S. J. Bobanović L. K. Murrell-Lagnado R. D. (2002) Identification of a non-canonical tyrosine-based endocytic motif in an ionotropic receptor. J. Biol. Chem. 277, 35378–35385 12105201 10.1074/jbc.M204844200

[25] Allsopp R. C. Evans R. J. (2011) The intracellular amino terminus plays a dominant role in desensitization of ATP-gated P2X receptor ion channels. J. Biol. Chem. 286, 44691–44701 22027824 10.1074/jbc.M111.303917 PMC3247974

[26] Roberts J. A. Allsopp R. C. El Ajouz S. Vial C. Schmid R. Young M. T. Evans R. J. (2012) Agonist binding evokes extensive conformational changes in the extracellular domain of the ATP-gated human P2X1 receptor ion channel. Proc. Natl. Acad. Sci. U.S.A. 109, 4663–4667 22393010 10.1073/pnas.1201872109 PMC3311380

[27] Jones G. Willett P. Glen R. C. Leach A. R. Taylor R. (1997) Development and validation of a genetic algorithm for flexible docking. J. Mol. Biol. 267, 727–748 9126849 10.1006/jmbi.1996.0897

[28] Case D. A. Cheatham T. E. 3rd Darden T. Gohlke H. Luo R. Merz K. M. Jr. Onufriev A. Simmerling C. Wang B. Woods R. J. (2005) The Amber biomolecular simulation programs. J. Comput. Chem. 26, 1668–1688 16200636 10.1002/jcc.20290 PMC1989667

[29] Evans R. J. Lewis C. Buell G. Valera S. North R. A. Surprenant A. (1995) Pharmacological characterization of heterologously expressed ATP-gated cation channels (P2X purinoceptors). Mol. Pharmacol. 48, 178–183 7544432

[30] Fujiwara Y. Kubo Y. (2006) Regulation of the desensitization and ion selectivity of ATP-gated P2X_2_ channels by phosphoinositides. J. Physiol. 576, 135–149 16857707 10.1113/jphysiol.2006.115246 PMC1995631

[31] Xiong K. Stewart R. R. Weight F. F. Li C. (2004) Role of extracellular histidines in antagonist sensitivity of the rat P2X_4_ receptor. Neurosci. Lett. 367, 197–200 15331152 10.1016/j.neulet.2004.06.008

[32] Hülsmann M. Nickel P. Kassack M. Schmalzing G. Lambrecht G. Markwardt F. (2003) NF449, a novel picomolar potency antagonist at human P2X_1_ receptors. Eur. J. Pharmacol. 470, 1–7 12787824 10.1016/s0014-2999(03)01761-8

[33] Hausmann R. Rettinger J. Gerevich Z. Meis S. Kassack M. U. Illes P. Lambrecht G. Schmalzing G. (2006) The suramin analog 4,4′,4″,4‴[-(carbonylbis(imino-5,1,3-benzenetriylbis(carbonylimino)))tetra-kis-benzenesulfonic acid (NF110) potently blocks P2X_3_ receptors: subtype selectivity is determined by location of sulfonic acid groups. Mol. Pharmacol. 69, 2058–2067 16551782 10.1124/mol.106.022665

[34] Braun K. Rettinger J. Ganso M. Kassack M. Hildebrandt C. Ullmann H. Nickel P. Schmalzing G. Lambrecht G. (2001) NF449: a subnanomolar potency antagonist at recombinant rat P2X1 receptors. Naunyn Schmiedebergs Arch. Pharmacol. 364, 285–290 11521173 10.1007/s002100100463

